# A prospective pilot clinical trial evaluating the utility of a dynamic near-infrared imaging device for characterizing suspicious breast lesions

**DOI:** 10.1186/bcr1837

**Published:** 2007-12-18

**Authors:** Ronald X Xu, Donn C Young, Jimmy J Mao, Stephen P Povoski

**Affiliations:** 1 Biomedical Engineering Department, The Ohio State University, Columbus, OH 43210, USA; 2 Center for Biostatistics, Arthur G. James Cancer Hospital and Richard J. Solove Research Institute and Comprehensive Cancer Center, The Ohio State University, Columbus, OH 43210, USA; 3 ViOptix, Inc., 44061-B Warm Springs Boulevard, Fremont, CA 94538, USA; 4 Section of Surgical Oncology of the Department of Surgery, Arthur G. James Cancer Hospital and Richard J. Solove Research Institute and Comprehensive Cancer Center, The Ohio State University, Columbus, OH 43210, USA

## Abstract

**Introduction:**

Characterizing and differentiating between malignant tumors, benign tumors, and normal breast tissue is increasingly important in the patient presenting with breast problems. Near-infrared diffuse optical imaging and spectroscopy is capable of measuring multiple physiologic parameters of biological tissue systems and may have clinical applications for assessing the development and progression of neoplastic processes, including breast cancer. The currently available application of near-infrared imaging technology for the breast, however, is compromised by low spatial resolution, tissue heterogeneity, and interpatient variation.

**Materials and methods:**

We tested a dynamic near-infrared imaging schema for the characterization of suspicious breast lesions identified on diagnostic clinical ultrasound. A portable handheld near-infrared tissue imaging device (P-Scan; ViOptix Inc., Fremont, CA, USA) was utilized. An external mechanical compression force was applied to breast tissue. The tissue oxygen saturation and hemoglobin concentration were recorded simultaneously by the handheld near-infrared imaging device. Twelve categories of dynamic tissue parameters were derived based on real-time measurements of the tissue hemoglobin concentration and the oxygen saturation.

**Results:**

Fifty suspicious breast lesions were evaluated in 48 patients. Statistical analyses were carried out on 36 out of 50 datasets that satisfied our inclusion criteria. Suspicious breast lesions identified on diagnostic clinical ultrasound had lower oxygenation and higher hemoglobin concentration than the surrounding normal breast tissue. Furthermore, histopathologic-proven malignant breast tumors had a lower differential hemoglobin contrast (that is, the difference of hemoglobin concentration variability between the suspicious breast lesion and the normal breast parenchyma located remotely elsewhere within the ipsilateral breast) as compared with histopathologic-proven benign breast lesions.

**Conclusion:**

The proposed dynamic near-infrared imaging schema has the potential to differentiate benign processes from those of malignant breast tumors. Further development and refinement of the dynamic imaging device and additional subsequent clinical testing are necessary for optimizing the accuracy of detection.

## Introduction

Breast cancer remains the leading cause of newly diagnosed cases of cancer among women in the United States, and the second leading cause of cancer deaths [1]. In this regard, the development and refinement of more innovative tools for analyzing the structural and physiologic characteristics of breast tissue to more precisely characterize and differentiate malignant tumors, benign tumors, and normal breast tissue becomes increasingly important.

Current methods of breast screening have several limitations. First, only approximately 20% of suspicious breast lesions seen on mammography are ultimately proven malignant on diagnostic breast biopsy [2]. Second, more dense breast tissue results in decreased sensitivity for detecting suspicious lesions on mammography [3]. Third, considerable debate still exists within the medical community with regards to the effectiveness of clinical breast examination, as well as its lack of performance consistency and standardization [4]. Fourth, breast self-examination has not yet definitively been shown to be associated with an overall reduction in breast cancer mortality [5].

The most commonly used modalities for breast imaging are the mammogram, ultrasound, and magnetic resonance imaging [3]. These modalities primarily delineate the morphologic and structural characteristics of breast tissue. Functional and physiologic properties of the breast can be characterized by such tools as dynamic contrast-enhanced magnetic resonance imaging [6] and positron emission tomography [7]. These modalities for breast imaging, however, are not universally available and are highly cost prohibitive. In this regard, it remains a priority to develop low-cost, portable, noninvasive innovative imaging tools for structural and better functional characterization of the breast.

Near-infrared (NIR) diffuse optical imaging and spectroscopy is capable of measuring multiple physiologic parameters of biological tissue systems and may have clinical applications for assessing the development and progression of neoplastic processes, including breast cancer [8-11]. In comparison with traditional structural imaging modalities (that is, mammogram, ultrasound, and magnetic resonance imaging), NIR imaging reveals only functional properties of biological tissues without anatomical characterization (that is, low spatial resolution). Two major physiologic parameters measured by NIR imaging and useful in the assessment of tumor-bearing tissues are tissue blood oxygen saturation ([S_t_O_2_]) and total hemoglobin concentration ([Hbt]). Alterations in the local microvasculature and the metabolism of developing malignant processes as compared with the normal surrounding tissues may be detectable by NIR diffuse optical imaging and spectroscopy prior to detection of any recognizable structural changes. For example, malignant tissues may have lower oxygen saturation due to imbalanced oxygen supply and uptake and increased blood volume due to angiogenesis [12-14].

Furthermore, tumor hypoxia is a common characteristic of locally advanced malignancies and has been shown to be associated with a diminished therapeutic response and with disease progression [15]. Tumor hypoxia results from an imbalance between oxygen delivery and oxygen consumption [16]. On the one hand, oxygen delivery is impaired by structural abnormalities present in the tumor microvasculature. These structural abnormalities can result in numerous functional impairments within the tumor microenvironment, including capillary and vascular permeability [17], interstitial hypertension [18], and increased flow resistance. On the other hand, altered tumor cell metabolism, resulting in an elevated metabolic rate, can also contribute to tumor hypoxia [19]. The association of hypoxia with tumor development and progression suggests that continuous monitoring of tumor oxygenation and metabolism can play an important role in assessing the development and progression of malignant processes.

NIR has been used previously for measuring various breast tissue characteristics, such as tissue absorbance, the scattering coefficient, oxygen saturation, the hemoglobin concentration, the lipid content, and the water content [20-26]. Measurable differences between malignant breast lesions and normal breast tissue for these particular breast tissue characteristics can vary by as much as 75%, 23%, 50%, and 14%, respectively [23]. Nevertheless, the menopausal status of the patient can lead to significant intersubject variations in these breast tissue characteristics by up to 200%, 16%, 180, and 50%, respectively [22]. Furthermore, even for the same subject, spatial variation in the location measured within the breast [24] and the phase of the menstrual cycle [25] can produce significant variation in these breast tissue characteristics. These breast tissue characteristics can vary by up to 16%, 1%, 14%, and 5%, respectively, depending on the measurement position [24], and can vary by up to 54%, 15%, 43%, and 64%, respectively, depending on the phase of the menstrual cycle [25]. These potentially large variations in tissue physiologic properties seen in breast tissue imply that it is not appropriate to use absolute values of these tissue parameters.

Investigators have therefore begun to examine various dynamic imaging techniques for characterizing tissue properties [27-33]. The use of such dynamic imaging schemas introduces external dynamics stimuli, such as physiologic stimuli, chemical stimuli, or mechanical stimuli, to the biological tissue system in order to allow one to measure relative changes in tissue physiologic properties. By subtracting the tissue baseline measurement from the dynamic response, the potential influence of tissue heterogeneity and interpatient variation can be minimized.

We have previously developed and tested a handheld NIR probe for dynamic imaging and characterization of biological tissue systems *in vivo *[29,34]. In the current prospective clinical trial, we have applied this technology to the evaluation of suspicious breast lesions. This schema measures the relative changes of tissue physical and physiologic properties in response to an external compression stimulus. We hypothesize that the tumor oxygen dynamics in response to the external stimulus may provide important information regarding tissue viscoelasticity, metabolic demand, and vascular perfusion that may ultimately help to identify malignancy.

## Materials and methods

### Equipment

**Figure 1:**
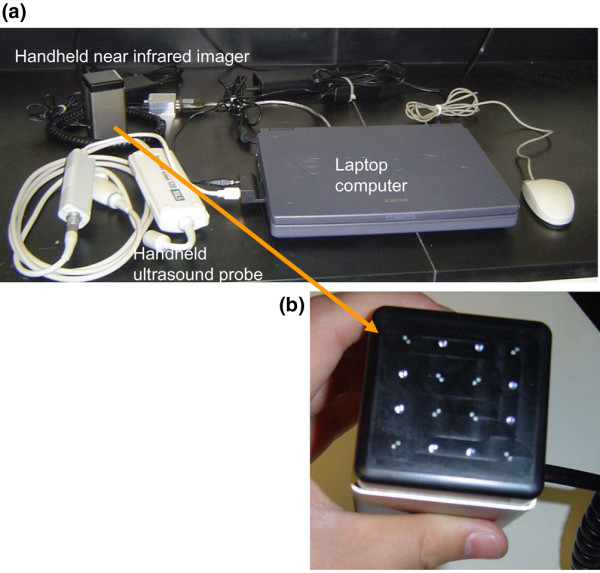
System setup for the dynamic imaging system. **(a) **System setup for dynamic imaging of suspicious breast lesions: the dynamic imaging system consists of a handheld near-infrared imager, a laptop computer, and a handheld ultrasound probe. **(b) **Close view of the handheld near-infrared imager.

**Figure 2:**
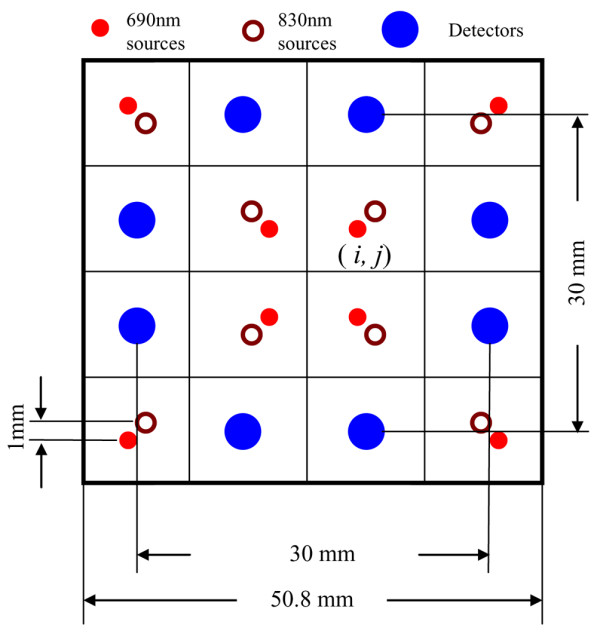
Source and detector configurations on the surface of the P-Scan near-infrared imager (not to scale). Sixteen sources and eight detectors are placed on the surface of the near-infrared imager, forming a 4 × 4 matrix. The overall size of the probe matrix is 50.8 mm × 50.8 mm. (*i*, *j*), pixels at the intersection of the *i*th row and the *j*th column. Tissue blood oxygen saturation ([S_t_O_2_]) and total hemoglobin concentration ([Hbt]) values were calculated at each pixel (*i*, *j*).

As previously described [31,32,34], a mobile experimental apparatus was developed that consists of a laptop computer, a portable clinical ultrasound system (Terason 2000; Teratech, Burlington, MA, USA), and a continuous wave-type, portable NIR tissue imager (P-Scan; ViOptix Inc., Fremont, CA, USA). The current system setup is shown in Figure 1. The portable clinical ultrasound system was used to locate the suspicious breast lesion for appropriate positioning of the portable NIR tissue imager. The NIR imager is a cuboid device measuring 5.5 cm × 5.5 cm × 10.2 cm in dimensions. The imager has a multilayer structure, consisting of a rigid aluminum outer shell, a telescoping insert, and a black Delrin sensor head. Sixteen light sources (eight sources for 690 nm and eight sources for 830 nm) and eight detectors were placed in a 3 cm × 3 cm matrix on the black Delrin sensor head, as shown in Figure 2. Light emission and detection were synchronized by a PIC microprocessor with a 12-bit A/D. The compression force was simultaneously measured by a miniature load cell embedded inside the sensor head. The system acquired 66 measurements (32 measurements of light reflectance at each wavelength and two measurements of compression force) within 500 milliseconds, and reconstructed the two-dimensional image of tissue oxygenation ([S_t_O_2_]) and hemoglobin concentration ([Hbt]) at a sampling rate of two frames per second.

In the past, various NIR devices have been developed for breast cancer detection [10]. These devices fall into three categories: continuous-wave devices, time-domain devices, and frequency-domain devices. A continuous-wave device illuminates the tissue with a steady or low-frequency light source. The tissue absorption property is calculated based on the attenuation of the reflectance intensity with the fixed input of a reduced scattering coefficient or a differential path length factor [35,36]. A time-domain device detects tissue optical properties with depth resolution by sending short laser pulses of femtoseconds to picoseconds into biological tissue and recording the time-of-flight and the amplitude of photons [37,38]. A frequency-domain device sinusoidally modulates at a high frequency from 100 MHz to 1,000 MHz. By measuring AC attenuation, DC attenuation, and the phase shift of the resulting diffuse photon density wave, one can retrieve both absorption and scattering properties of biological tissue with accuracy and with resistance to ambient noises [39].

The device we utilized in the present clinical trial is a continuous-wave device [34]. It differs from other continuous-wave devices in that all the optoelectronic components are integrated into a handheld unit without fiber optical connections. Another unique feature is that the device embeds a compression load cell that enables the feedback control of the compression load and the real-time monitoring of the pressure-induced tissue optical property changes. Compared with time-domain and frequency-domain devices, this device has the advantages of low cost, simplicity, and real-time image-processing capability. Owing to the limited number of wavelengths (690 nm and 830 nm) used in our device, however, only oxyhemoglobin and deoxyhemoglobin concentrations are detectable, assuming that these two chromophores are the major sensitive chromophores in the NIR region. In contrast, other researchers have used more wavelengths to retrieve additional information regarding water, lipid concentrations, and other chromophore concentrations [21,23].

### Study design

The present trial was an unblinded, pilot study to test the clinical feasibility of a dynamic NIR imaging device. The study protocol was approved by the Ohio State University Comprehensive Cancer Center Scientific Review Committee (Protocol Number OSU-04117) and by the Cancer Institutional Review Board of the Arthur G. James Cancer Hospital and Richard J. Solove Research Institute and Comprehensive Cancer Center of Ohio State University (IRB number 2005C005).

Clinical data collection was performed at JamesCare (Dublin, OH, USA), our comprehensive breast health services facility of the Arthur G. James Cancer Hospital and Richard J. Solove Research Institute and Comprehensive Cancer Center of Ohio State University. Patients were eligible to participate in the study if they were 18 years of age or older, were not pregnant, and had a suspicious breast lesion (Breast Imaging Reporting and Data System 4 or 5) that was identifiable on ultrasound (either on both mammogram and ultrasound or on ultrasound alone). Eligible patients were informed of the investigational nature of the study protocol and were required to read, agree to, and sign a statement of informed consent prior to participation.

The breast dynamic NIR imaging session for each eligible subject was arranged to take place immediately after a diagnostic clinical ultrasound was performed, but prior to a scheduled ultrasound-guided breast biopsy procedure. During the dynamic NIR imaging session, each subject was placed in a supine position, with her ipsilateral arm placed above her head and her ipsilateral upper torso tilted slightly upward. First, the Terason 2000 ultrasound probe was used to detect the location and the shape of the suspicious lesion. Subsequently, the P-Scan imager was then placed over the area of the suspicious breast lesion for continuous data collection.

**Figure 3:**
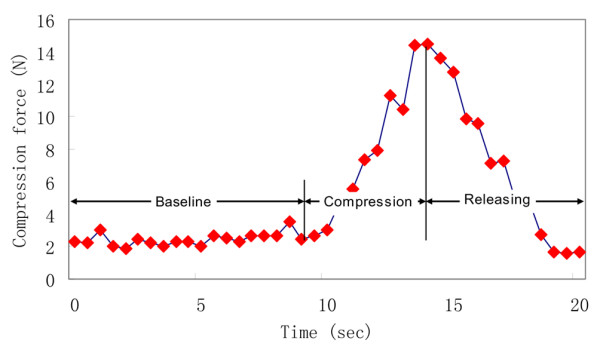
Typical compression force profile detected during a single dynamic loading cycle. A typical compression force profile detected during a single dynamic loading cycle when the operator compresses the P-Scan device on breast tissue and gradually releases it. The compression profile was divided into three sections: baseline, compression, and releasing. Each test cycle started with a baseline measurement at a minor load of 2 N for about 10 seconds, followed by a ramped compression load up to 15 N during 5 seconds, and ended with a gradual releasing of the compression load during 5 seconds.

Each dynamic loading cycle lasted for a period of approximately 20 seconds, during which time a compression force (up to a maximum of 15 N) was gradually applied to and released from the breast tissue by the operator. The selection of 15 N as the maximal compression force is in compliance with Mammography Quality Standard Act regulation 900.12(e)(4). The 20-second compression frequency was estimated by taking into account the device's sampling rate, the operator's controllability, and the viscoelastic response of the biological tissue. Each 20-second cycle consisted of a 10-second baseline measurement, 5 seconds of gradual increasing compression (compression phase), and 5 seconds of gradually releasing compression (release phase). As the P-Scan imager was compressed on and gradually released from the breast tissue by the operator, the compression force was recorded dynamically, as shown in Figure 3.

**Figure 4:**
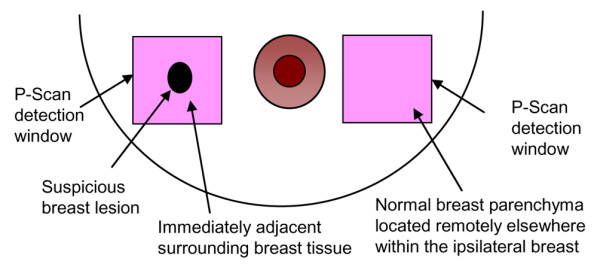
Schematic depiction of the P-Scan breast imaging technique. For each patient, a dynamic compression profile was generated for both the area of the suspicious breast lesion (which includes the suspicious breast lesion itself that was identified on ultrasound and the immediately adjacent surrounding breast tissue) and the normal reference tissue (representing normal breast parenchyma located remotely elsewhere within the ipsilateral breast).

For each patient, such a dynamic compression profile was generated for both the area of the suspicious breast lesion (which includes the suspicious breast lesion itself that was identified on ultrasound and the immediately adjacent surrounding breast tissue) and the normal reference tissue (representing normal breast parenchyma verified on diagnostic clinical ultrasound and located remotely elsewhere within the ipsilateral breast). This is schematically shown in Figure 4. An ultrasound-guided breast biopsy was performed on each patient after the initial dynamic NIR imaging session was completed.

From March 2005 to August 2005, a total of 48 patients with a suspicious breast lesion seen on ultrasound were recruited into this prospective pilot clinical trial, of which two patients had bilateral suspicious breast lesions. Data were therefore collected on a total of 50 suspicious breast lesions with reference baseline measurements determined from normal breast parenchyma located remotely elsewhere within the ipsilateral breast. The average depth of the 50 suspicious breast lesions from the skin surface was 1.15 cm (range, 0.36–2.66 cm; standard deviation, ± 0.50 cm). Of these 50 suspicious breast lesions, 14 had a calculated cuboidal volume estimate (based on the three dimensions of the lesion recorded on ultrasound) of greater than 3.375 cm^3 ^(approximating a three-dimensional lesion size of 1.5 cm × 1.5 cm × 1.5 cm). These 14 suspicious breast lesions were excluded from the data analysis based on the following rationale: since the P-Scan imager has an active detection area less than 3 cm × 3 cm, a lesion with a cross-sectional area that is greater than 1.5 cm × 1.5 cm would result in compromised contrast between the lesion and the immediately adjacent surrounding breast tissue in two-dimensional [S_t_O_2_]/[Hbt] maps; and since the reference baseline measurements were taken on normal breast parenchyma located remotely elsewhere within the ipsilateral breast, a lesion with a cross-sectional area that is greater than 1.5 cm × 1.5 cm would increase the risk of interference between reference baseline measurements and lesion measurements. For these reasons, we derived tissue parameters and performed biostatistics analyses on 36 lesions only.

### Tissue parameters

For each set of measurements, [*S*_*t*_*O*_2_]_*i*,*j *_and [*Hbr*]_*i*,*j *_were reconstructed at each pixel (*i*, *j*) in Figure 2, where *i *= 1–4 and *j *= 1–4 [35]. The following tissue parameters were derived based on dynamic measurements of tissue [*S*_*t*_*O*_2_]_*i*,*j *_and [*Hbr*]_*i*,*j*_. Parameters 1–6 are applicable for both baseline measurements and measurements at peak compression loads.

Parameter 1. Averaged tissue [S_t_O_2_]:

(1) $$\bar{\left[S_{t}O_{2}\right]}=\frac{1}{16}\sum_{i=1}^{4}\sum_{j=1}^{4}{\left[S_{t}O_{2}\right]}_{i,j}$$

This parameter was averaged over 16 pixels (4 × 4 matrix) of a single detection window. The parameter was calculated both at baseline (no compression) and at peak compression load.

Parameter 2. Averaged tissue [Hbt]:

(2) $$\bar{\left[Hbt\right]}=\frac{1}{16}\sum_{i=1}^{4}\sum_{j=1}^{4}{\left[Hbt\right]}_{i,j}$$

This parameter was averaged over 16 pixels (4 × 4 matrix) of a single detection window. The parameter was calculated both at baseline (no compression) and at peak compression load.

Parameter 3. Relative [S_t_O_2_] contrast:

(3) $$\delta [S_{t}O_{2}]=\sqrt{\sum_{i=1}^{4}\sum_{j=1}^{4}{\left({\left[S_{t}O_{2}\right]}_{i,j}-\bar{\left[S_{t}O_{2}\right]}\right)}^{2}/{\left(4\bar{\left[S_{t}O_{2}\right]}\right)}^{2}}$$

This parameter calculated the standard deviation of [S_t_O_2_] values in a 4 × 4 matrix. It was an indicator of [S_t_O_2_] heterogeneity over the region of detection.

Parameter 4. Relative [Hbt] contrast:

(4) $$\delta [Hbt]=\sqrt{\sum_{i=1}^{4}\sum_{j=1}^{4}{\left({\left[Hbt\right]}_{i,j}-\bar{\left[Hbt\right]}\right)}^{2}/{\left(4\bar{\left[Hbt\right]}\right)}^{2}}$$

This parameter calculated the standard deviation of [Hbt] values in a 4 × 4 matrix. It was an indicator of [Hbt] heterogeneity over the region of detection.

Parameter 5. Differential [S_t_O_2_]:

(5) $$\bar{\bar{\left[S_{t}O_{2}\right]}}=\left({\bar{\left[S_{t}O_{2}\right]}}^{T}-{\bar{\left[S_{t}O_{2}\right]}}^{R}\right)/{\bar{\left[S_{t}O_{2}\right]}}^{T}$$

This parameter calculated the relative $\bar{\left[S_{t}O_{2}\right]}$ difference between the suspicious breast lesion (denoted '*T*' for tumor) and the normal breast parenchyma located remotely elsewhere within the ipsilateral breast (denoted '*R*' for reference).

Parameter 6. Differential [Hbt]:

(6) $$\bar{\bar{\left[Hbt\right]}}=\left({\bar{\left[Hbt\right]}}^{T}-{\bar{\left[Hbt\right]}}^{R}\right)/{\bar{\left[Hbt\right]}}^{T}$$

This parameter calculated the $\bar{\left[Hbt\right]}$ difference between the suspicious breast lesion and the normal breast parenchyma located remotely elsewhere within the ipsilateral breast.

Parameter 7. Differential [S_t_O_2_] contrast:

(7) *D *[*S*_*t*_*O*_2_] = *δ*[*S*_*t*_*O*_2_]_*q *_^*T *^- *δ*[*S*_*t*_*O*_2_]_*q *_^*R*^   *q *= *B*, *P*

This parameter compared [S_t_O_2_] heterogeneity between the suspicious breast lesion and the normal breast parenchyma located remotely elsewhere within the ipsilateral breast. ('*B*' represents baseline measurements and '*P*' represents measurements at the peak compression load.)

Parameter 8. Differential [Hbt] contrast:

(8) *D *[*Hbt*] = *δ*[*Hbt*]_*q *_^*T *^- *δ*[*Hbt*]_*q *_^*R*^   *q *= *B*, *P*

This parameter compared [Hbt] heterogeneity between the suspicious breast lesion and the normal breast parenchyma located remotely elsewhere within the ipsilateral breast.

Parameter 9. Variation in differential [S_t_O_2_]:

(9) $$\Delta [S_{t}O_{2}]={\bar{\bar{\left[S_{t}O_{2}\right]}}}_{B}-{\bar{\bar{\left[S_{t}O_{2}\right]}}}_{P}$$

This parameter evaluated how differential [S_t_O_2_] changes in response to compression force.

Parameter 10. Variation in differential [Hbt]:

(10) $$\Delta [Hbt]={\bar{\bar{\left[Hbt\right]}}}_{B}-{\bar{\bar{\left[Hbt\right]}}}_{P}$$

This parameter evaluated how differential [Hbt] changes in response to compression force.

Parameter 11. Differential variations of [S_t_O_2_]:

(11) *DD *[*S*_*t*_*O*_2_] = Δ[*S*_*t*_*O*_2_]^*T *^- Δ[*S*_*t*_*O*_2_]^*R*^

This parameter evaluated how the [S_t_O_2_] values of the tumor and the normal breast parenchyma located remotely elsewhere within the ipsilateral breast respond differently to the compression load.

Parameter 12. Differential variations of [Hbt]:

(12) *DD *[*Hbt*] = Δ[*Hbt*]^*T *^- Δ[*Hbt*]^*R*^

This parameter evaluated how the [Hbt] values of the tumor and the normal breast parenchyma located remotely elsewhere within the ipsilateral breast respond differently to the compression load.

### Statistical analyses

The software program SPSS 14.0 for Windows (SPSS, Inc., Chicago, IL, USA) was used for all statistical analyses. First, the tissue parameters were analyzed by comparing all suspicious breast lesions identified on ultrasound (designated 'tumor') versus all of the normal reference tissues (representing the normal breast parenchyma located remotely elsewhere within the ipsilateral breast; designated 'reference'). Second, the tissue parameters were then analyzed by comparing all histopathologic-proven benign lesions (designated 'benign') versus all histopathologic-proven malignant lesions (designated 'malignant').

Since the ramped compression profile introduced coupled, transient reactions in tissue structure and tissue [S_t_O_2_]/[Hbt], we analyzed only the baseline measurements and measurements at the peak compression load. Since none of the data deviated from normality using the Kolmogorov Smirnov test, we compared 'tumor' versus 'reference tissue' using paired t tests and we compared 'benign' versus 'malignant' using unpaired t tests. A paired t test was used for the 'tumor' versus 'reference tissue' comparisons since, in each instance, these represented paired observations that were collected from the same patient. An unpaired t test (two-independent-sample t test) was used for the 'benign' versus 'malignant' comparisons since these represented two independent groups of unequal sample sizes. Since a large number of different comparisons of the multiple tissue parameters were analyzed, only resulting *P *< 0.01 values were considered statistically significant.

## Results

A total of 36 out of 50 suspicious breast lesions had a calculated cuboidal volume estimate ≤ 3.375 cm^3 ^and were included in the current analyses. Of these 36 suspicious breast lesions, 22 showed benign histopathology (designated 'benign') and 14 showed malignant histopathology (designated 'malignant'). Table 1 presents the histopathology for these 36 suspicious breast lesions.

**Table 1:** Histopathology findings for the 36 suspicious breast lesions

Lesion	Histopathology findings	*n*
Benign tumors (*n *= 22)	Fibroadenoma	10
	Fibrocystic changes	4
	Ruptured cyst and fat necrosis	4
	Intraductal papilloma	3
	Sclerosing adenosis	1
Malignant tumors (*n *= 14)	Invasive ductal carcinoma	12
	Medullary carcinoma	1
	Intracystic papillary carcinoma	1

Table 2 presents the paired *t*-test results for all 36 suspicious breast lesions ('tumor') versus their normal reference tissue ('reference'). First, 'tumor' differed from 'reference' in the averaged [S_t_O_2_] for both the baseline measurement (*P *< 0.001) and the compression peak measurement (*P *< 0.001). Second, 'tumor' differed from 'reference' in their averaged [Hbt] for both the baseline measurement (*P *< 0.001) and the compression peak measurement (*P *< 0.001). Third, 'tumor' differed from 'reference' by the relative [S_t_O_2_] contrast at the compression peak (*P *= 0.01). In this regard, 'tumor' showed larger [S_t_O_2_] contrast than 'reference', indicating a larger heterogeneity in oxygen distribution for suspicious breast lesions versus normal reference tissues.

**Table 2:** Paired *t*-test results comparing tissue parameters for the 36 suspicious breast lesions ('tumor') versus normal breast parenchyma within the ipsilateral breast ('reference')

Tissue parameter	Tissue type	Mean ± standard deviation	*P *value
Averaged [S_t_O_2_] – baseline (%)	Tumor/reference	88.7 ± 8.9/92.8 ± 5.7	*<0.001*
Averaged [Hbt] – baseline (μM)	Tumor/reference	11.0 ± 6.0/9.0 ± 5.0	*<0.001*
Relative [S_t_O_2_] contrast – baseline (%)	Tumor/reference	3.6 ± 3.7/2.3 ± 1.1	0.03
Relative [Hbt] contrast – baseline (%)	Tumor/reference	15.3 ± 8.8/12.8 ± 7.0	0.04
Averaged [S_t_O_2_] – compression peak (%)	Tumor/reference	87.7 ± 10.0/91.7 ± 7.1	*<0.001*
Averaged [Hbt] – compression peak (μM)	Tumor/reference	10.0 ± 6.0/8.0 ± 4.0	*<0.001*
Relative [S_t_O_2_] contrast – compression peak (%)	Tumor/reference	4.0 ± 3.7/2.8 ± 1.7	*0.01*
Relative [Hbt] contrast – compression peak (%)	Tumor/reference	15.5 ± 9.8/13.9 ± 10.0	0.2
Variation in differential [S_t_O_2_] (%)	Tumor/reference	-0.5 ± 1.7/-0.4 ± 0.9	0.96
Variation in differential [Hbt] (%)	Tumor/reference	-0.2 ± 4.6/-1.2 ± 4.6	0.23

[Hbt], total hemoglobin concentration; [S_t_O_2_], tissue blood oxygen saturation.

Significant *P *values are italicized.

**Figure 5:**
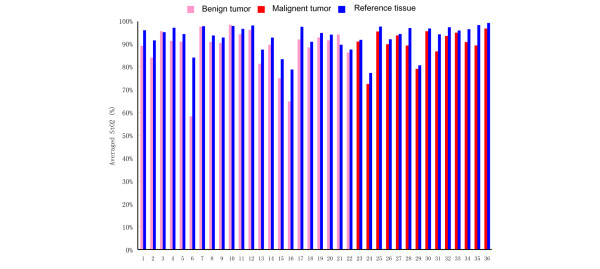
Averaged tissue blood oxygen saturation measurements for benign tumors, malignant tumors, and normal reference tissues. *x *axis, number of patients; *y *axis, averaged tissue blood oxygen saturation ([S_t_O_2_]) measurements. Pink bars, [S_t_O_2_] measurements on the suspicious breast lesions (tumor); blue bars, [S_t_O_2_] measurements on the normal surrounding breast parenchyma (reference). Patients 1–22 were diagnosed with benign tumors (patients 1 and 2, atypical intraductal papilloma; patients 3–5, fibrocystic changes; patient 6, fibrocystic changes with radial scar; patients 7–15, fibroadenoma; patient 16, fibroadenoma with lactational change; patient 17, intraductal papilloma; patients 18–21, ruptured cyst and fat necrosis; and patient 22, sclerosing adenosis). Patients 23–36 were diagnosed with malignant tumors (patient 23, intracystic papillary carcinoma; patient 24–35, invasive ductual carcinoma; and patient 36, medullary carcinoma). For each patient, averaged [S_t_O_2_] values were calculated on both the tumor tissue and the reference tissue based on [S_t_O_2_] measurements by the P-Scan probe.

**Figure 6:**
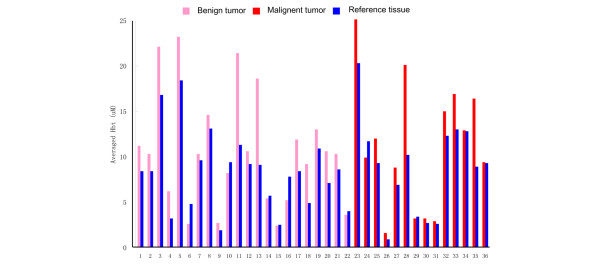
Averaged total hemoglobin concentration measurements for benign tumors, malignant tumors, and their normal reference tissues. *x *axis, number of patients; *y *axis, averaged total hemoglobin concentration ([Hbt]) measurements. Pink bars, [Hbt] measurements on the suspicious breast lesions (tumor); blue bars, [Hbt] measurements on the normal surrounding breast parenchyma (reference). Patients 1–22 were diagnosed with benign tumors (patients 1 and 2, intraductal papilloma with mild atypia; patients 3–5, fibrocystic changes; patient 6, fibrocystic changes with radial scar; patients 7–15, fibroadenoma; patient 16, fibroadenoma with lactational change; patient 17, intraductal papilloma; patients 18–21, ruptured cyst and fat necrosis; and patient 22, sclerosing adenosis). Patients 23–36 were diagnosed with malignant tumors (patient 23, intracystic papillary carcinoma; patients 24–35, invasive ductual carcinoma; and patient 36, medullary carcinoma). For each patient, averaged [Hbt] values were calculated on both the tumor tissue and the reference tissue based on [Hbt] measurements by the P-Scan probe.

**Figure 7:**
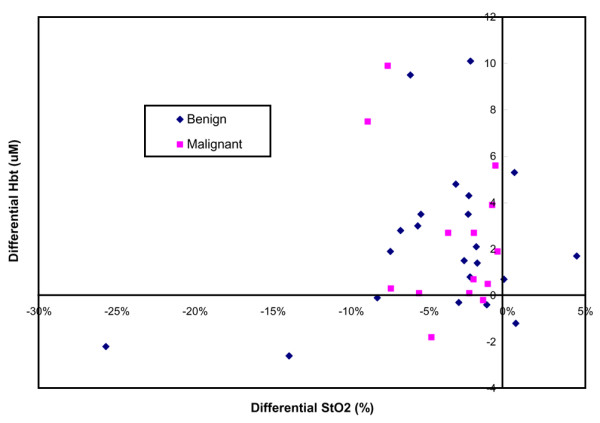
Differential tissue blood oxygen saturation and total hemoglobin concentration measurements for benign and malignant tumors. Plot based on the dataset shown in Figures 4 and 5. *x *axis, differential tissue blood oxygen saturation ([S_t_O_2_]) determined by subtracting the [S_t_O_2_] measurement of the suspicious breast lesion (tumor) from that of the normal surrounding breast parenchyma (reference); *y *axis, differential total hemoglobin concentration ([Hbt]) determined by subtracting the [Hbt] measurement of the suspicious breast lesion (tumor) from that of the normal surrounding breast parenchyma (reference).

Despite these differences in tissue [S_t_O_2_] and [Hbt] for suspicious breast lesions (including both 'benign' tumors and 'malignant' tumors) and for their normal reference tissue, it remains very difficult to derive generalized criteria that are applicable for distinguishing 'benign' versus 'malignant'. This difficulty, best exemplified in Figures 5 and 6, is clearly due to tissue heterogeneity and interpatient variations. Figure 5 plots the averaged [S_t_O_2_] for all 'benign' tumors, 'malignant' tumors, and their normal reference tissue. Figure 6 plots the averaged [Hbt] for all 'benign' tumors, 'malignant' tumors, and their normal reference tissue. From these plots, it is generally very difficult to differentiate specific tissue types based on the averaged [S_t_O_2_] versus the averaged [Hbt]. As is shown in Figure 7, however, if differential [S_t_O_2_] and differential [Hbt] values are plotted for each individual suspicious breast lesion (including both 'benign' and 'malignant'), most of the datapoints localize within the same quadrant. This indicates that any given suspicious breast lesion ('tumor') tends to display a lower oxygenation and a higher hemoglobin concentration as compared with its surrounding normal reference tissue.

Table 3 summarizes the unpaired *t*-test comparisons for multiple features (including patient age, tumor volume, and numerous tissue parameter comparisons) derived from the 22 'benign' tumors and the 14 'malignant' tumors. 'Benign' tumors and 'malignant' tumors differed from one another by age, differential [Hbt] contrast at baseline, and differential [Hbt] contrast at compression peak (*P *= 0.006, *P *= 0.008 and *P *= 0.004, respectively). While 'benign' tumors were found in younger patients, displayed higher differential [Hbt] contrast at baseline, and displayed higher differential [Hbt] contrast at compression peak, 'malignant' tumors were found in older patients, displayed lower differential [Hbt] contrast at baseline, and displayed lower differential [Hbt] contrast at compression peak. Furthermore, mechanical compression further enhanced the differences between 'benign' tumors and 'malignant' tumors for differential [Hbt] contrast.

**Table 3:** Unpaired *t*-test results comparing multiple features (including patient age, tumor volume, and numerous tissue parameter comparisons) for the 22 'benign' tumors and the 14 'malignant' tumors

Parameter	Tissue type	Mean ± standard deviation	*P *value
Age (years)	Benign/malignant	44.0 ± 13.9/57.6 ± 13.6	*0.006*
Tumor volume (cm^3^)	Benign/malignant	1.0 ± 0.9/1.4 ± 0.7	0.13
Tumor tissue [S_t_O_2_] – baseline (%)	Benign/malignant	87.9 ± 10.1/89.9 ± 6.8	0.51
Tumor tissue [Hbt] – baseline (μM)	Benign/malignant	11.0 ± 6.0/11.0 ± 7.0	0.77
Reference tissue [S_t_O_2_] – baseline (%)	Benign/malignant	92.4 ± 5.3/93.5 ± 6.5	0.57
Reference tissue [Hbt] – baseline (μM)	Benign/malignant	8.0 ± 4.0/9.0 ± 5.0	0.74
Differential [S_t_O_2_] – baseline (%)	Benign/malignant	-6.1 ± 10.0/-4.1 ± 3.3	0.47
Differential [Hbt] – baseline (%)	Benign/malignant	12.1 ± 32.3/17.8 ± 19.7	0.56
Tumor [S_t_O_2_] contrast – baseline (%)	Benign/malignant	4.1 ± 4.6/2.8 ± 1.2	0.34
Tumor [Hbt] contrast – baseline (%)	Benign/malignant	16.6 ± 8.1/13.2 ± 9.7	0.27
Reference tissue [S_t_O_2_] contrast – baseline (%)	Benign/malignant	2.3 ± 1.1/2.3 ± 1.2	0.88
Reference tissue [Hbt] contrast – baseline (%)	Benign/malignant	11.7 ± 5.6/14.4 ± 8.7	0.27
Differential [S_t_O_2_] contrast – baseline (%)	Benign/malignant	1.7 ± 4.1/0.5 ± 1.4	0.32
Differential [Hbt] contrast – baseline (%)	Benign/malignant	4.8 ± 6.3/-1.2 ± 6.2	*0.008*
Tumor tissue [S_t_O_2_] – compression peak (%)	Benign/malignant	87.2 ± 11.4/88.6 ± 7.8	0.7
Tumor tissue [Hbt] – compression peak (μM)	Benign/malignant	10.0 ± 6.0/11.0 ± 7.0	0.58
Reference tissue [S_t_O_2_] – compression peak (%)	Benign/malignant	91.4 ± 6.9/92.2 ± 7.6	0.76
Reference tissue [Hbt] – compression peak (μM)	Benign/malignant	7.0 ± 4.0/8.0 ± 5.0	0.62
Differential [S_t_O_2_] – compression peak (%)	Benign/malignant	-6.2 ± 12.8/-4.2 ± 4.7	0.59
Differential [Hbt] – compression peak (%)	Benign/malignant	13.0 ± 33.7/22.6 ± 22.1	0.35
Tumor [S_t_O_2_] contrast – compression peak (%)	Benign/malignant	4.3 ± 4.2/3.6 ± 2.8	0.56
Tumor [Hbt] contrast – compression peak (%)	Benign/malignant	16.4 ± 7.9/14.0 ± 12.5	0.48
Reference [S_t_O_2_] contrast – compression peak (%)	Benign/malignant	2.8 ± 1.4/2.8 ± 2.2	0.97
Reference [Hbt] contrast – compression peak (%)	Benign/malignant	12.2 ± 6.1/16.7 ± 13.9	0.18
Differential [S_t_O_2_] contrast – compression peak (%)	Benign/malignant	1.6 ± 3.4/0.8 ± 1.7	0.45
Differential [Hbt] contrast – compression peak (%)	Benign/malignant	4.3 ± 6.3/-2.7 ± 6.8	*0.004*
Variation in differential [S_t_O_2_] (%)	Benign/malignant	0.1 ± 4.5/0.2 ± 2.1	0.96
Variation in differential [Hbt] (%)	Benign/malignant	-0.9 ± 12.0/-4.9 ± 8.3	0.29
Variation in differential [S_t_O_2_] – tumor (%)	Benign/malignant	-0.3 ± 1.4/-0.8 ± 2.1	0.41
Variation in differential [Hbt] – tumor (%)	Benign/malignant	0.2 ± 4.2/-0.8 ± 5.2	0.54
Variation in differential [S_t_O_2_] – reference (%)	Benign/malignant	-0.4 ± 0.6/-0.5 ± 1.3	0.79
Variation in differential [Hbt] – reference (%)	Benign/malignant	-0.4 ± 3.5/-2.3 ± 5.9	0.24
Differential variations of [S_t_O_2_] – compression peak (%)	Benign/malignant	0.1 ± 1.7/-0.3 ± 1.2	0.44
Differential variations of [Hbt] – compression peak (%)	Benign/malignant	0.6 ± 4.7/1.5 ± 4.4	0.57

[Hbt], total hemoglobin concentration; [S_t_O_2_], tissue blood oxygen saturation.

Significant *P *values are italicized.

## Discussion

Solid tumors are generally associated with reduced oxygen saturation (due to hypoxia) [14-16,19] and with increased hemoglobin concentration (due to unregulated angiogenesis) [12,17,18]. NIR diffuse optical imaging and spectroscopy has been used for noninvasive characterization of tumor-induced tissue parameter changes [10]. Previous researchers have demonstrated that breast tumors (both breast cancer and fibroadenoma) typically show relatively lower oxygen saturation and higher hemoglobin concentration in comparison with those of reference breast tissues [9,23,40-42]. The pilot clinical trial reported in the present manuscript demonstrated consistent results in comparison with findings reported by previous researchers.

Despite the observed differences between tumor and normal tissue in terms of hemoglobin concentration and oxygen saturation, clinical application of NIR technology into the arena of breast cancer detection has not yet been realized. One of the major obstacles is the difficulty to derive generalized criteria for characterizing tissue differences that would ultimately allow one to distinguish 'benign' findings versus 'malignant' tumors within the breast. The difficulty comes from the significant tissue heterogeneities and interpatient variations that may exceed the characteristic difference between benign and malignant tumors [22-25]. To overcome this difficulty, some investigators studied tissue dynamic characteristics in response to physiologic, chemical, and mechanical stimuli [27-30,32]. Other investigators derived relative diagnostic criteria by calculating differential oxygen and hemoglobin parameters between the tumor and the reference tissue in the contralateral, cancer-free breast [20].

To minimize the influence of tissue heterogeneity and the interpatient variation, we developed a dynamic breast imaging schema. By defining relative tissue parameters and studying pressure-induced changes in these parameters, we hope to derive more effective detection criteria that are less sensitive to tissue heterogeneities and interpatient variations. Although some tissue parameters defined in this paper are not yet fully utilized due to the instrumentation limitations, the dynamic imaging concept discussed in the present paper paves the way for a low-cost, portable, reproducible imaging method for breast cancer detection.

The results of the current prospective pilot clinical trial confirmed the clinical potential of the currently evaluated dynamic NIR imaging schema. Statistically significant differences were observed between 'tumor' and its surrounding normal reference tissue for averaged [S_t_O_2_] and [Hbt] levels. Generally speaking, 'tumor' shows higher [Hbt] (probably due to angiogenesis) and lower [S_t_O_2_] (probably due to hypoxia) than the normal surrounding breast parenchyma. Likewise, among all 'tumors', differences between 'benign' tumors and 'malignant' tumors were demonstrated in terms of differential hemoglobin contrast, indicating that 'benign' lesions display higher heterogeneity in terms of hemoglobin concentration than do 'malignant' lesions. No difference was observed, however, between 'benign' lesions and 'malignant' lesions in terms of other tissue parameters, such as averaged [S_t_O_2_] and [S_t_O_2_] contrast.

From the results of the current prospective pilot clinical trial, it is evident that several technical limitations exist. Such limitations of the currently evaluated dynamic NIR imaging schema will need to ultimately be overcome in order to create a more clinically relevant imaging schema.

The first technical issue of the current prospective pilot clinical trial relates to the use of ramped compression (that is, gradual increase or decrease of the compression load). Such a ramped compression profile may not be the most ideal mechanism for providing the dynamic stimulus. During ramped compression, hemodynamic changes are intrinsically coupled with tissue viscoelastic deformation that is induced by the action of ramped compression. This makes the resultant quantitative analysis of such events quite difficult. For this particular reason, and as previously mentioned in Materials and methods, our statistical analyses only considered two datapoints of each dynamic loading cycle (that is, the baseline measurement and the compression peak measurement). Additionally, the 5-second ramped compression load used in this protocol was not sufficient to result in significant tissue oxygenation changes, as would be observed during tissue ischemia secondary to vascular occlusion. In our protocol, therefore, the [S_t_O_2_] fluctuations measured during compression were not the result of tissue ischemia, but were instead the result of additional tissue heterogeneity induced by relative changes in the position of a given suspicious breast lesion and its adjacent normal reference tissue.

We propose two potential solutions to resolve these issues with ramped compression. First, we can separate tissue deformation from tissue physiologic changes by simultaneous structural and functional imaging using an integrated NIR and ultrasound imaging system. Second, we can replace the ramped compression profile with a stepped compression profile (that is, sudden increase or decrease of the compression load). Such a stepped compression profile would uncouple the tissue mechanical reaction (that is, transient tissue deformation) from the physiologic reaction (that is, tissue ischemia). As the result of using an integrated imaging system and a stepped compression profile, we expect to see characteristic differences in other tissue parameters that will ultimately help to better differentiate 'malignant' from 'benign' lesions.

The second technical issue of the current prospective pilot clinical trial relates to the instrumentation of the NIR imaging system utilized. In general, NIR imaging systems fall into three technical platforms, based on differences in the time dependence of the excitation source intensity and the detection mechanism [10]. These technical platforms include continuous-wave devices, time-domain devices, and frequency-domain devices [10].

In the current prospective pilot clinical trial, we utilized a continuous wave NIR system (that is, the P-Scan imager). The intrinsic limitation of such a continuous-wave system is that the scattering coefficient cannot be explicitly resolved, and therefore differential path length factors or reduced scattering coefficients have to be assumed [43]. In our current prospective pilot clinical trial, constant reduced scattering coefficients were used (${\mu^{\prime }}_{s\_690}$ = 5/cm, ${\mu^{\prime }}_{s\_830}$ = 4/cm). The mismatch between the assumed scattering coefficient and the actual scattering coefficient can introduce measurement bias in [S_t_O_2_] and [Hbt]. In our current prospective pilot clinical trial, a relatively higher [S_t_O_2_] and a relatively lower [Hbt] were observed as compared with [S_t_O_2_] and [Hbt] measurements recorded by other NIR imaging systems, such as a frequency domain device [40]. Despite the above measurement bias, the use of a continuous-wave device may still preserve its clinical applicability secondary to its simplistic design and its low cost. Furthermore, in our continuous-wave dynamic NIR imaging schema, we derived only relative, differential tissue parameters based on [S_t_O_2_] and [Hbt] measurements, regardless of their resultant bias in absolute values. In this regard, the influence of the scattering coefficient mismatch would be further reduced.

A final technical issue regarding the instrumentation of the NIR imaging system utilized in the current prospective pilot clinical trial is related to the limited image resolution. This limited image resolution is the result of an insufficient number of source-detector pixel positions for optical measurements and the poor coregistration between sequential NIR and ultrasound imaging. The intrinsic problem of our limited imaging resolution is related to high scattering and exponential attenuation of light in biological tissues. To improve upon the limited imaging resolution of our current NIR imaging system instrumentation, we plan to implement a step-motorized scanning mechanism to increase the number of optical measurement positions. Additionally, we plan to utilize an integrated NIR and ultrasound imaging system for simultaneous image acquisition during each dynamic loading cycle in order to monitor tumor deformation and changes in tumor depth. We believe the implementation of such an integrated system will allow us to more precisely characterize specific differences between benign and malignant breast lesions.

## Conclusion

The current prospective pilot clinical trial – utilizing a dynamic NIR imaging device for characterizing breast tissue differences – demonstrated that suspicious breast lesions ('tumor') had a lower [S_t_O_2_] and a higher [Hbt] than its normal reference tissues ('reference'), and that 'benign' lesions can be distinguished from 'malignant' lesions based on differential [Hbt] contrast. Although further technology development and additional clinical testing are necessary to improve the detection sensitivity and specificity, the present prospective pilot clinical trial demonstrates the potential of such a dynamic NIR imaging device for identifying breast tissue differences that may ultimately be useful as a noninvasive modality for distinguishing benign processes from those of breast cancer.

## Abbreviations

[Hbt] = total hemoglobin concentration; NIR = near infrared; [S_t_O_2_] = tissue blood oxygen saturation.

## Competing interests

The P-Scan imager used for the clinical trial is a precommercial prototype of ViOptix Inc. RXX, SPP, and DCY have no competing interests. JJM is an employee of ViOptix, Inc.

## Authors' contributions

RXX was the principle investigator responsible for the dynamic NIR imaging system development, for clinical data collection, and for data analyses. SPP was the clinical investigator supervising patient recruitment and data collection. DCY performed all biostatistical analyses. JJM provided engineering support for the P-Scan imager and helped to prepare the clinical protocol. RXX and SPP were responsible for writing and editing all portions of the manuscript. All the authors read and approved the final version of the manuscript.
